# Microbially-Induced Exosomes from Dendritic Cells Promote Paracrine Immune Senescence: Novel Mechanism of Bone Degenerative Disease in Mice

**DOI:** 10.14336/AD.2022.0623

**Published:** 2023-02-01

**Authors:** Ranya Elsayed, Mahmoud Elashiry, Yutao Liu, Ana C. Morandini, Ahmed El-Awady, Mohamed M. Elashiry, Mark Hamrick, Christopher W. Cutler

**Affiliations:** ^1^Department of Periodontics, Dental College of Georgia at Augusta University, GA, USA.; ^2^Department of Cellular Biology and Anatomy, Augusta University, GA, USA.; ^3^Department of Oral Biology and Diagnostic Sciences, Dental College of Georgia, Augusta University, GA, USA.; ^4^Department of Endodontics, Faculty of Dentistry, Ain Shams University, Cairo Egypt.

**Keywords:** dendritic cells, SASP, exosomes, immune senescence, periodontitis, *porphyromonas gingivalis*

## Abstract

As the aging population grows, chronic age-related bone degenerative diseases become more prevalent and severe. One such disease, periodontitis (PD), rises to 70.1% prevalence in Americans 65 years and older. PD has been linked to increased risk of other age-related diseases with more serious mortality and morbidity profiles such as Alzheimer’s disease and cardiovascular disease, but the cellular and biological mechanisms remain unclear. Recent *in vitro* studies from our group indicate that murine dendritic cells (DCs) and T cells are vulnerable to immune senescence. This occurs through a distinct process involving invasion of DCs by dysbiotic pathogen *Porphyromonas gingivalis* (Pg) activating the senescence associated secretory phenotype (SASP). Exosomes of the Pg-induced SASP transmit senescence to normal bystander DC and T cells, ablating antigen presentation. The biological significance of these findings *in vivo* and the mechanisms involved were examined in the present study using young (4-5mo) or old (22-24mo) mice subjected to ligature-induced PD, with or without dysbiotic oral pathogen and injection of Pg-induced DC exosomes. Senescence profiling of gingiva and draining lymph nodes (LN) corroborates role of advanced age and PD in elevation of senescence biomarkers beta galactosidase (SA-β-Gal), p16 ^INK4A^ p21^Waf1/Clip1^, IL6, TNFα, and IL1β, with attendant increase in alveolar bone loss, reversed by senolytic agent rapamycin. Immunophenotyping of gingiva and LN revealed that myeloid CD11c+ DCs and T cells are particularly vulnerable to senescence *in vivo* under these conditions. Moreover, Pg-induced DC exosomes were the most potent inducers of alveolar bone loss and immune senescence, and capable of overcoming senescence resistance of LN T cells in young mice. We conclude that immune senescence, compounded by advanced age, and accelerated by oral dysbiosis and its induced SASP exosomes, plays a pivotal role in the pathophysiology of experimental periodontitis.

Periodontitis (PD) is a chronic inflammatory disease of aging or inflammaging that is particularly prevalent in those 65 yrs. and older [1]. PD is also comorbid with other more serious age-related inflammatory diseases [2] typified by enhanced peripheral inflammation and impaired immune-surveillance [3]. These intriguing observations suggest a common underlying mechanism that may account for such comorbidities. With advanced age comes an increase in accumulation of senescent cells [4]. Senescent osteocytes have been identified in the alveolar bone of old mice [5]. Senescent macrophages are documented in gingiva of diabetic mice and ameliorated by the senolytic agent metformin [6] while rapamycin is reported to inhibit age-related alveolar bone loss in mice [7].

Cellular senescence (CS) is described as a stable cell cycle arrest accompanied by distinct phenotypic and functional alterations and a proinflammatory secretome in response to a variety of cell stressors. CS is considered a physiologic defense mechanism against tumorigenesis entailing a complex inflammatory process occurring in both replicative and terminally differentiated cells [4]. Halted proliferation of damaged or dysfunctional cells and resistance to apoptosis that accompany extreme DNA/cellular damage is a feature of CS [8]. Further observed in CS is increased chronic inflammation, impairment of wound healing, and ineffective immune responses to microbes [9] and tumor antigens [3]. CS has been identified in several age-related diseases such as atherosclerosis, diabetes, lung disease and many others [8]. Removal of senescent cells reduces age-related diseases and increases longevity in mice [10]. Premature senescence due to chronic exposure to cell stressors can also occur in the young [11]. CS stressors include genotoxins such as anti-cancer drug doxorubicin, ROS, as well as danger associated molecular patterns (DAMPs) [4].

Immune cells are vulnerable to senescence, with functional incompetence reported in monocytes, macrophages (reviewed in [12]), T cells (reviewed in [13]), and dendritic cells (DCs) [12, 14]. Senescent immune cells are apoptosis resistant, functionally impaired, and express the senescence-associated secretory phenotype (SASP). Our laboratory has recently reported that the dysbiotic oral pathogen *Porphyromonas gingivalis* (Pg) infects DCs and activates the SASP *in vitro*. The SASP consists of secreted cytokines (e.g., IL-1β, IL-6, IL-8) and exosomes. Exosomes, nano-sized extracellular vesicles (EVs) that originate in the endocytic pathway, [15] were first discovered from sheep reticulocytes in 1987 [16]. Since then, the ubiquity of exosomes in all tissues and body fluids including saliva have been recognized. Exosomes contain molecular cargo that is transmitted, in paracrine, to bystander cells, impairing their function [17].

A hallmark of the established PD lesion is influx of dendritic cells (DCs) clustered with CD4+ T cells in lamina propria [18]. Unregulated DC activation in periodontal tissues promotes Th17-mediated bone loss [17]. Dysbiotic pathogen Pg is found in DCs in the PD lesion and in the bloodstream [19]. Only recently, we identified Pg as an initiator of senescence in DCs in vitro, involving cellular invasion and secretion of exosomes [14]. Soluble inflammatory cytokines such as TNFa, IL-1 α/β, IL-6, IL-8, IL-12 are released locally in PD lesions [20], and especially IL-1β through activation of the inflammasome [21]. Tissue destruction feeds further growth of oral pathogens [22],. Elevated basal pro-inflammatory cytokine levels and reduced capacity to undergo maturation in response to lipopolysaccharide (LPS) production were elevated in aged DCs [14, 23]. The role of immune senescence in the pathogenesis and susceptibility to PD remains largely unexplored.

Growing evidence supports a critical role of exosomes in immune senescence, inflammaging and age-related diseases [24]. The purpose of the present study was to investigate the role of advanced age and oral dysbiosis in induction of immune senescence *in vivo* in mice and how the associated SASP exosomes influence inflammatory bone loss in a murine PD model.

## MATERIALS AND METHODS

### Ethics statement

The Institutional Animal Care and Use Committee (IACUC) of Augusta University (protocol# 2013-0586) approved all experimental procedures.

### Animal study design

PD in humans is most prevalent in individuals aged 65+ [1] (= 20+ mouse months) and entails dysbiotic oral infection with *P. gingivalis* (Pg) and other species. In this study young (4-5mo) and old (22-24mo) C57BL6 mice (n=36) were subjected to ligature placement on maxillary second molar using silk 5-0. The ligature was left in place in all mice for the whole experimental period (21 days). The contralateral molar tooth in each mouse was left un-ligated to serve as baseline internal control for alveolar bone volume measurements. Young (n=18) and old (n=18) animals were randomly divided into 3 groups (n=6-7 per group; 3 males and 3 females). LIPD Group: received only 2% CMC vehicle by oral gavage and PBS/0.5% DMSO vehicle injection on the ligation site. LIPD + Pg Group: received Pg in 2% CMC by oral gavage and PBS 0.5% DMSO injection on the ligation site. LIPD +Pg +Rap Group: received Pg in 2% CMC by oral gavage and rapamycin in 0.5%DMSO. Local injection of 10 ul of rapamycin 10 μM [25] (R8781)(Sigma Aldrich, St Louise, MO, USA) suspended in 0.5% DMSO was performed into palatal gingiva using Hamilton micro syringe with 33 gauge and 10 μL loading capacity (Hamilton, NV, USA) at day -2, day 0 and day 2 in relation to ligature placement day and then every 3 days during the experimental period. *P. gingivalis* 381 (Pg) was maintained anaerobically in (10%, H2, 10% CO2, and 80% N2) in a Coy lab vinyl anaerobic chamber (Coy Laboratory Products, Inc., Grass Lake, MI) at 37°C in Wilkins-Chalgren anaerobe broth. Bacterial cells were maintained until mid-log phase. Bacterial colony forming units (CFU) were calculated based on a spectrophotometer OD 660 reading of 0.11, previously reported to equal 5x10^7 CFU. [26] 10^9 CFU of Pg were suspended in 2%CMC (Carboxymethyl cellulose) in sterile PBS and administered to animals via oral gavage every other day for 21 days. At the end of the 21 days, animals were euthanized, and hemimaxillae harvested for assessment of alveolar bone loss by microcomputed tomography (micro-CT). Gingival tissues were harvested from the same animals and further processed for flow cytometry, confocal microscopy, and mRNA analysis. Draining lymph nodes were excised, cells isolated and immunolabeled to be analyzed by flow cytometry.

### Micro-CT imaging and bone parameter analysis

The hemi-maxillae were scanned using 1272 Skyscan System (Bruker, Belgium) at 9.5 μm image pixel size with 0.25 mm Al filter, 0.2 rotation step and frame averaging set at 3. 3-D reconstruction was done using NRecon software (Skyscan) and 3-D analysis of alveolar bone loss around ligated maxillary second molar was performed using CTAn software (Skyscan) as previously described [17, 27]. Briefly, the region of interest (ROI) was standardized in all samples by taking the cemento-enamel junction of the mesial surface of second molar as a start reference point till the root apex (40 slices) in an occluso-apical direction and from the distal end of maxillary 1st molar till the mesial end of maxillary 3rd molar in mesio-distal direction. Greyscale images of the volume of interest (VOI) were converted to binary images with a threshold set to 80-255. Residual bone volume (BV, μm3) around the second upper molars was quantified, and 3-D models were generated with teeth. The un-ligated contralateral molar tooth in each mouse served as internal control for alveolar bone volume measurements[17].

### SA-β-Gal staining and confocal microscopy

Cell Event Senescence Green flow cytometry assay kit (Thermofisher Scientific, Waltham MA, USA) was used according to the manufacturer’s instruction. Briefly frozen gingival tissue sections were allowed to thaw at room temperature and then rinsed with PBS (no Ca, no Mg). Tissue sections were then fixed with 4% paraformaldehyde (PFA) for 10 minutes at room temperature followed by washing 3x in PBS to remove the fixative solution. For labelling CD11c^+^ dendritic cells, tissues were permeabilized with 0.1%Triton x-100 for 10 min followed by rinsing 3x in PBS. Then slides were incubated overnight with anti-mouse CD11c antibody N418 (Invitrogen; Cat#MA11C5) at a dilution of 1:20 at 4° C or Cytokeratin 14 polyclonal antibody (Proteintech; Cat#10143-1-AP) at a dilution of 1:100. Lack of primary antibody was used to exclude nonspecific binding of the secondary antibody. Next day, slides were washed 3x with PBS and then incubated with secondary antibody; Goat anti Hamster IgG TRITC (Invitrogen; Cat#PA1-28667) at a dilution of 1:1000, and Goat anti Rabbit IgG TRITC (Invitrogen, Cat#T-2769) at a dilution of 1:500 for 1 hour at room temperature with subsequent washing 3x with PBS (5 min each). Slides were then incubated with Cell Event Senescence Green probe (Thermofisher Scientific, Waltham MA, USA) at 1:500 dilution for 2 hours at 37°C with no CO2. Slides were then washed with PBS, 2% FBS and nuclear counterstaining was performed using DAPI mounting medium; ProLong Gold Antifade Mountant (Invitrogen, Thermofisher Scientific, Waltman MA, USA). Multiple random photomicrographs were taken in the lamina propria of each gingival tissue at 20x objective lens using Zeiss 780 upright confocal microscope (Carl Zeiss AG, Oberkochen, Germany) and quantification was done blindly using automated Image-J software (https://imagej.nih.gov/ij/) with a set threshold based on the negative control. The quantification was confirmed by individual cell count performed by a blinded observer (Supplementary Fig. 8).

### Flow cytometry and antibodies

Gingival tissues from maxillae were removed and treated with Collagenase type II (2 mg/mL) and DNase I (1 mg/mL) (both from Sigma Aldrich, St Louise, MO, USA) solution in PBS plus 2% FCS for 30min at 37°C in a shaker bath. 0.25μL of 0.5M EDTA per 2 mL sample were added and incubated for an additional 15minutes, followed by centrifugation at 4°C, 400 g for 8 min in PBS with 2% FBS. Supernatants were removed, cells were suspended in PBS with 2% FBS and filtered through a 70-μm cell strainer. Lymph nodes were excised, and cells were isolated by mincing lymph nodes over a cell strainer placed on top of petri dish with 5 mL PBS-1mMEDTA- 2%FBS. Using the flat end of a sterile syringe plunger, cells were macerated through the strainer, followed by 2X centrifugation at 4°C, 300 g for 5 min in PBS-2% FBS. For intracellular staining of cytokines, cells from lymph nodes were stimulated *ex vivo* using CD3/CD28 antibodies (Thermofisher scientific; Cat#16-0032-82/Cat#16-0281-85). Staining of single cell suspension from gingival tissues and lymph nodes was performed on ice, with Flow Cytometry Staining Buffer (Thermofisher scientific). Blocking of FC receptors (FCR) was achieved with mouse FcR blocking reagent (Miltenyi Biotec) for 15minutes on ice and protected from light, followed by addition of fluorophore-conjugated antibody at recommended concentration on ice for 30 minutes, after which cells were washed, re-suspended in flow cytometry buffer. Intracellular staining was performed after fixation and permeabilization using fixation/permeabilization buffer set (eBioscience, Thermofisher Scientific, Waltham MA, USA) according to the manufacturer’s protocol. Cells were incubated with conjugated antibodies in 1x permeabilization buffer followed by washing and resuspension in flow cytometry buffer. Data was acquired and analyzed using Novocyte Quanteon flow cytometer machine and software (Agilent Technologies, CA, USA). Antibodies used: anti-mouse CD11c APC; clone N418 (Affymetrix, eBioscience, Thermofisher Scientific, Waltham MA, USA; Cat#17-0114-82)., anti-mouse MHCII Viogreen; clone M5/114.15.2, (Miltenyi Biotech Auburn, CA, USA; Cat#130-119-122), anti-mouse IL-1B PE; clone PE, Clone NJTEN3 (Affymetrix, eBioscience, Thermofisher Scientific, Waltham MA, USA; Cat#12-7114-82)., anti-mouse TNF alpha FITC; clone MP6-XT22 (Invitrogen, Thermofisher Scientific, Waltham MA, USA; Cat#11-7321-82), anti-mouse IL-6 eFluor 450; clone MP5-20F3 (Invitrogen, Thermofisher Scientific, Waltham MA, USA; Cat#48-7061-82), anti-mouse CD11c eFluor 450 ; clone N418 (Affymetrix, eBioscience, Thermofisher Scientific, Waltham MA, USA; Cat#48-0114-82), anti-mouse CD4 Vioblue; clone GK1.5 (Invitrogen, Thermofisher Scientific, Waltham MA, USA; Cat#48-0041-82), anti-mouse CD3e FITC; clone 145-2C11(Invitrogen, Thermofisher Scientific, Waltham MA, USA; Cat#11-0031-82), anti-mouse CD28 APC; clone 37.51 (Invitrogen, Thermofisher Scientific, Waltham MA, USA; Cat#17-0281-82), anti-mouse/rat IL-17A PE; clone eBio17B7 (Thermofisher Scientific, Waltham MA, USA; Cat#12-7177-81), anti-mouse CD57 PE; clone HNK-1(Santa Cruz Biotechnology; Cat#SC-81633 PE).

### SA-β-gal flow cytometry staining of CD11c positive DCs in gingival tissues

Cells were isolated from gingival tissues by enzymatic digestion as described above. Then single cell suspension was used to detect SA-β-gal in DCs by flow cytometry. Cell Event Senescence Green flow cytometry assay kit (Thermofisher Scientific, Waltham MA, USA) was used according to the manufacturer’s instruction. Briefly DCs were first stained for cell surface marker, anti-mouse CD11c APC; clone N418 (Invitrogen; Cat#17-0114-82) using regular staining protocol discussed in detail in the above section. After the last wash, cells were fixed with 4% paraformaldehyde (PFA) for 10 minutes at room temperature followed by washing to remove the fixative solution. Cells were then incubated with Cell Event Senescence Green probe (Thermofisher Scientific, Waltham MA, USA) at 1:500 dilution for 2 hours at 37°C with no CO2. Cells were then washed with PBS, 2% FBS, resuspended in FACS staining buffer and data acquired and analyzed by MACSQuant analyzer machine and MACSQuantify software (Miltenyi Biotech, Auburn, CA, USA).

### Real time PCR

Gingival tissues were kept in RNA latter and stored in -80 degree until the time of RNA isolation. At the time of isolation, gingival tissues were allowed to thaw in room temperature and immediately moved to RLT lysis buffer; QIAGEN RNeasy mini kit (Qiagen, Inc., Valencia, CA, USA). Then gingival tissue samples were suspended in the lysis buffer and minced into small pieces using a sharp lancet, and vortexed thoroughly. Tissues were disrupted and homogenized using TissueLyser LT (Qiagen, Inc., Valencia, CA, USA) at 25 oscillations for 5 min. Then samples were centrifuged at maximum speed for 3 min and supernatants transferred to a new tube. Then total RNA was isolated using QIAGEN RNeasy mini kit (Qiagen, Inc., Valencia, CA, and USA) according to the manufacturer’s protocol. RNA purity and concentration were measured using Nanodrop (NanoDrop 1000 UV-VIS Spectrophotometer Software Ver.3.8.1, Thermofisher Scientific). Ratio of 260/280 of 2.0 was considered adequate for analysis. Reverse transcription to cDNA was performed using the High-Capacity cDNA Reverse Transcription Kit (Applied Biosystem, Thermofisher Scientific, Waltham MA, USA) in a total reaction of 20 μL. Quantitative real-time PCR was performed using TaqMan fast advanced master mix (Applied Biosystem, Thermofisher Scientific, Waltham MA, USA) and TaqMan Gene Expression assay (Applied Biosystem, Foster City, CA, USA) specific for: p21WAF cdkn1a (Mm042 05640_g1), p16(INK4a) cdkn2a (Mm00494449_m1) and IL1B (Mm00434228_m1), internal control beta actin (Actb Mm02619580_g1). RT-PCR was run using StepOnePlus Real-Time PCR System. Calculation of relative mRNA expression was performed using delta-delta CT and presented as relative fold-change to the control group.

### Generation and culture of Murine bone marrow-derived dendritic cells (BMDCs)

Bone marrow cells were isolated from C57BL/6 mice (6-12 weeks old) as previously described [14]. Briefly, cells were flushed from femurs and tibiae of mice under sterile conditions then allowed to differentiate in RPMI 1640 10% FBS with 20 ng/mL GM-CSF and 20 ng/mL IL-4 (Pepro Tech Pepro, NJ, USA) at a density of 1x10^6^ cells/mL and incubated at 5% CO2 and 37°C for 8 days. Culture media was changed every 2 days with addition of fresh growth factors and cells were harvested on day 6 and incubated for 2 days in EXO depleted complete media (by using EXO free FBS) to generate iDCs [17, 27].

### P.gingivalis bacterial culture and DCs infection

*P.gingivalis* 381 (Pg) was maintained anaerobically in (10%, H2, 10% CO2, and 80% N2) in a Coy lab vinyl anaerobic chamber (Coy Laboratory Products, Inc., Grass Lake, MI) at 37°C in Wilkins-Chalgren anaerobe broth. Bacterial cells were maintained until mid-log phase. Bacterial CFU were calculated based on a spectrophotometer OD 660 reading of 0.11, previously determined to equal 5x10^7 CFU [26]. Infection of DCs with *P.gingivalis* was performed as previously described [14]. Briefly, at day 6 of DC culture, cells were pulsed with *P.gingivalis* at a multiplicity of infection (MOI) of 10 for 48 hours. On day 8, cells were washed and incubated for 24 hrs. and on day 9, cells were isolated and, culture supernatants were collected for EXO purification.

### Exosome isolation and puriﬁcation

Exosome isolation was performed as previously described by our group [14, 17]. Briefly, supernatants from DC cultures underwent differential centrifugation (successive centrifugations at 500 g for (5 min), 2000g for (20 min), and 10,000 g for (30 min) to remove cells and debris. Then, ultrafiltration 2x with 0.2 µm and 2x with 100 kDA filters (to remove microvesicles and free proteins) and ultracentrifugation for 90 min at 120,000 g were performed. Then EXO pellets were washed with PBS and ultra-centrifuged 2x at 120,000 g for 90 min, and finally re-suspended in 100 µl of PBS, stored in -80 for further analysis and functional studies. Contamination with Pg outer membrane vesicle (OMV) was excluded by passing the exo on CD81/CD9/CD63+ magnetic bead column a previously shown by our group [14].

### Nanoparticle tracking analysis of DC-derived exosomes

Nanoparticle tracking analysis (NTA) by Zeta View PMX 110 (Particle Metrix, Meerbusch, Germany) was used for analysis of size, count, and distribution of nanoparticles in suspension, as previously described [14, 17]. Briefly, 10 µl of the sample was diluted in 1ml 1xPBS and loaded into the sample chamber, then size and concentration of the sample were automatically generated by the software (ZetaView 8.02.28).

### Electron microscopy

As described previously [14, 17] exosome sample was fixed in 4% paraformaldehyde in 0.1M cacodylate buffer PH 7.4 overnight. Five microliters (5µl) of suspended exosome preparation were applied to a carbon-Formvar coated 200 mesh nickel grid and allowed to stand 30 minutes. The excess sample was wicked off onto Whatman filter paper. Grids were floated exosome side down onto a 20 µl drop of 1M Ammonium Chloride for 30 minutes to quench aldehyde groups from the fixation step. Grids were floated on drops of blocking buffer (0.4% BSA in PBS) for 2 hours. Then rinsed 3X with PBS (5 minutes each). Grids were set up as follows and allowed to incubate in blocking buffer or the primary antibody (anti CD63) for 1 hour. Grids were floated on drops of 1.4 nm secondary antibody nanogold (Nanoprobes, Inc.) diluted 1:1000 in blocking buffer for 1 hour. Grids were rinsed 3 X 5 minutes each with DI. For visibility in the electron SEM), exosome sample was postfixed in 2% osmium tetroxide in NaCac buffer and dehydrated in ethanol. Then mounted on aluminum stubs and sputter-coated 6 minutes with gold-palladium (Anatech Hummer 6.2, Union City, CA). Exosomes were observed and imaged at 10 kV using an FEI XL30 scanning electron microscope (FEI, Hillsboro, OR).

### Exosome uptake in DCs and T cells in vivo in PD model

Dil-labeled exosomes were used to demostrate the uptake of exo *in vivo*, as previously described [17]. Briefly, exosomes derived from dendritic cells infected with Pg (PgDCexo) were labeled with Vybrant™ DiI Cell-Labeling Solution (Thermofisher) and injected in the palatal gingiva of mice. Mice were sacrificed 24 hours after injection, gingival tissues excised and frozen. Cryosections were then mounted on slides and stored in -80 for further processing. Frozen gingival tissue sections were allowed to thaw at room temperature and then rinsed with PBS (no Ca, no Mg). Tissue sections were then fixed with 4% paraformaldehyde (PFA) for 10 minutes at room temperature followed by washing 3x in PBS to remove the fixative solution. For labelling of CD11c^+^ dendritic cells and CD 4^+^ T cells, tissues were permeabilized with 0.1%Triton x-100 for 10 min followed by rinsing 3x in PBS. Then slides were incubated overnight at 4° C with anti-mouse CD11c antibody N418 (Invitrogen; Cat#MA11C5) at a dilution of 1:20 or antiCD4 (GK1.5) monoclonal antibody at a dil of 1:20 (Thermofisher; Cat# MA1-146). No primary antibody controls were used to exclude nonspecific binding of the secondary antibody. Next day, slides were washed 3x with PBS and then incubated with secondary antibody; Goat anti-Rat IgG, DyLight 488 (SA5-10018) and Goat anti-Hamster IgG, Alexa Fluor 488 (A-21110) (Thermofisher Scientific) for 1 hour at room temperature with subsequent washing 3x with PBS (5 min each). Then slides were mounted with DAPI, and images captured with Zeiss 780 upright confocal microscope (Carl Zeiss, AG, Oberkochen, Germany).

### Intragingival injection of PgDCexo in young animals

To study the effect of exosomes derived from *P.gingivalis*-infected dendritic cells (PgDCexo) on premature senescence induction and inflammatory alveolar bone loss, young (4-5 mo) C57/BL6 mice (n=24) were used subjected to ligature placement on the right maxillary second molar using silk 5-0. The ligature was left in place in all mice for the experimental period (8 days). The contralateral molar tooth (left side) in each mouse was left un-ligated to serve as baseline internal control for alveolar bone volume measurements. Animals were divided randomly into 4 groups (n=6), Group 1 (No exo): received only sham injection with vehicle, Group2 (imDCexo): received injection with exo derived from immature DCs with no infection, Group 3 (PgDCexo): received injection with exo derived from DCs infected with Pg, Group4 (PgDCexo +Rap): received injection with exo derived from DCs infected with Pg and rapamycin. Local injections of 10^8^ exo [17] ± 10μM rapamycin were performed into palatal gingiva using Hamilton micro syringe with 33 gauge and 10 μL loading capacity (Hamilton, NV, USA) at day -2, day 0, day 2 and day 4 in relation to ligature placement day [17] (study design found in Supplemental data (Supplementary Fig. 5)). On day 8, animals were euthanized, and hemi-maxillae harvested for assessment of alveolar bone loss by microcomputed tomography (micro-CT), gingival tissues were harvested and further processed for flow cytometry, confocal microscopy and mRNA analysis using qPCR. Draining lymph nodes were excised and cells isolated and immunolabeled to be analyzed by flow cytometry.

### Analysis of PBMCs from peripheral blood of young and old human subjects

Buffy coats from young (n=3, mean age 24+5.5) and old (n=3, mean age 68.3+2.8) healthy donors were obtained from Community Blood Center (periodontal status unknown). Buffy coats were further processed to get rid of residual RBCs by using FICOL density gradient separation. Then the buffy coats were collected and washed 2x and then suspended in FACS buffer for flow cytometry. Antibodies used: anti-human CD45R0 PerCP-eFluor 710; clone UCHL1 (ebioscience, Thermofisher Scientific, Waltham MA, USA; Cat#46-0457-41), anti-human CD27 eFluor 450; clone 0323 (ebioscience, Thermofisher Scientific, Waltham MA, USA; Cat#48-0279-42), anti-human IgD FITC; clone IA6-2 (ebioscience, Thermofisher Scientific, Waltham MA, USA; Cat#11-9868-42), anti-human CD57 PE Cyanine7; clone TB01(ebioscience, Thermofisher Scientific, Waltham MA, USA; Cat#25-0577-42), anti-human CD14 eFluor 450; clone 61D3 (ebioscience, Thermofisher Scientific, Waltham MA, USA; Cat#48-0149-42), anti-human CD3 PerCP Vio700; clone BW264/56 (Miltenyi Biotech Auburn, CA, USA; Cat #130-097-582), anti-human CD3 FITC; clone BW264/56 (Miltenyi Biotech Auburn, CA, USA; Cat #130-080-401), anti-human CD45RA VioGreen (Miltenyi Biotech Auburn, CA, USA; Cat#130-098-184), anti-human CD4 VioBlue; clone VIT4 (Miltenyi Biotech Auburn, CA, USA#130-098-163), anti-human CD16 VioGreen (Miltenyi Biotech Auburn, CA, USA; Cat#130-098-095), anti-human CD28 FITC; clone CD28.2 (ebioscience, Thermofisher Scientific, Waltham MA, USA; Cat#11-0289-42), anti-human CD8a PE; clone OKT8 (ebioscience, Thermofisher Scientific, Waltham MA, USA; Cat#12-0086-42), anti-human CD19 PE (BD Biosciences; Cat #555413), anti-human CD4 VioBlue (Miltenyi Biotech Auburn, CA, USA; Cat#130-099-683).

### Statistical analysis

Data was analyzed using GraphPad Prism 9 (GraphPad Software, La Jolla, CA). Data analysis was performed by two-way, one-way ANOVA, or student T test with significance defined as P < 0.05, and confidence level of 95% confidence interval followed by Tukey’s or Bonferroni post hoc multiple-comparisons test. Normal distribution assumption was evaluated using Shapiro-Wilk normality test. When normality assumptions were not met, an alternative non-parametric test was used accordingly. Data are expressed as mean ± standard deviation (SD) and experiments were repeated 2 times

## RESULTS

### Gingival senescence induction central to alveolar bone loss in response to local factors and exacerbated by advanced age

**Figure 1.**
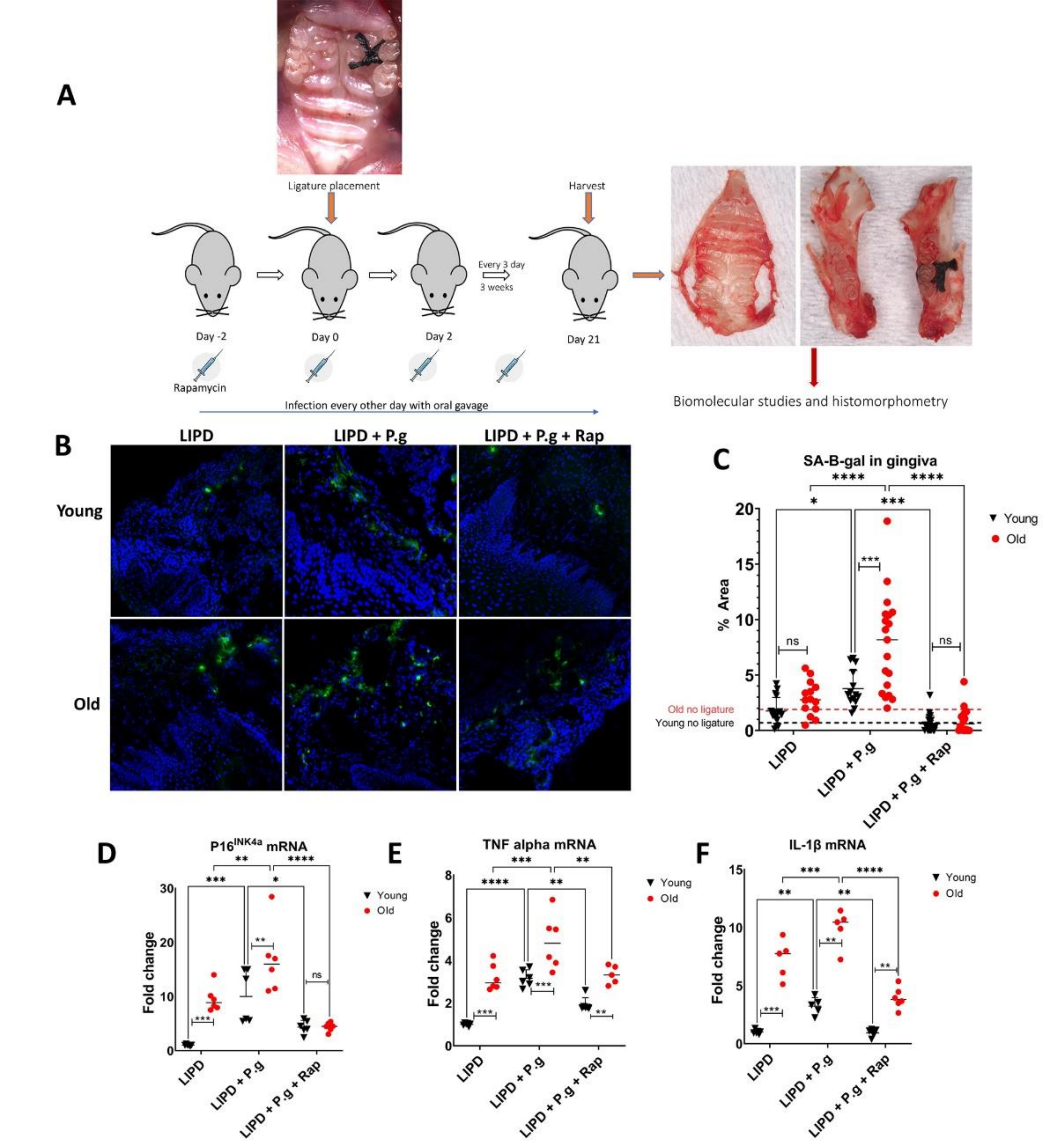
Cellular senescence profiling of gingiva. Gingiva of young and old mice (N=6-7 per group) subjected to ligature induced PD (LIPD), with or without Pg gavage and senolytic agent rapamycin (Rap), was stripped, and frozen sections were used for detection of SA-B-gal using fluorescent senescent B-gal probe, and gingival tissues were pooled to isolate RNA for mRNA expression analysis using qPCR. (A) A schematic diagram showing the animal study design. (B) Representative confocal microscopy images showing SA-B-gal expression in the gingiva (green) and counterstained with DAPI for nuclei. (C) Quantification of SA-B-gal expression plotted as % area of SA-B-gal^+^cells using image J software (N=4 per group with multiple random images taken per animal). (D, E, F) qPCR analysis showing relative mRNA expression (relative to young LIPD group) of p16 ^INK4A^, TNFa, and IL-1β in gingival tissues from young and old mice subjected to LIPD, with or without Pg gavage and senolytic agent Rap (N=6 per group, gingival tissues were pooled with n=6 technical replicates). Two-way ANOVA and Tukey post-hoc multiple comparison test used (*p<0.05, **p<0.001, ***p<0.0001).

**Figure 2.**
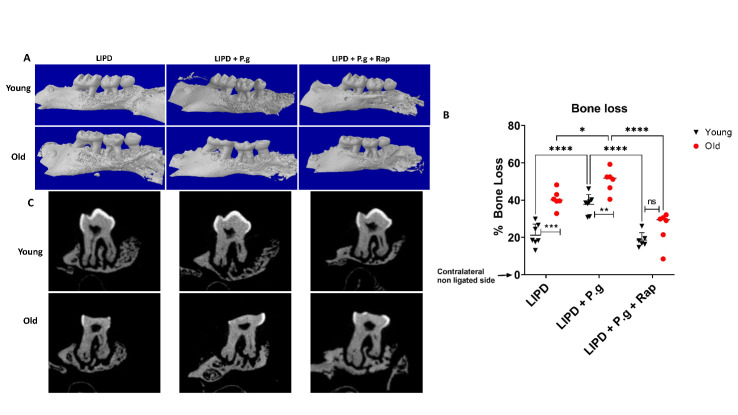
Alveolar bone loss as function of age and Pg-gavage. (A) Micro CT showing % bone loss in 3-D in young/old B.6 mice subjected to PD, with/without Pg gavage and rapamycin (Rap). All bone data normalized to contralateral non-ligature side of respective mouse.^20^ (B) Bar graph showing %bone loss (C) Cross-sections of molar root and alveolus are shown using microCT (N=6-7 per group, Two-way ANOVA and Tukey post-hoc multiple comparison test used (*p<0.05, **p<0.001, ***p<0.0001).

Preliminary studies established baseline measures of bone volume and gingival senescence biomarker SA-b gal expression in young and old control mice without addition of local exogenous factors. Evidence for significant loss of alveolar bone levels (Supplementary Fig.1 A and B) and increased gingival expression of senescence biomarker SA-b gal (Supplementary Fig. 1C and D), as a function of mouse age, necessitated the use of internal controls in each mouse to assess role of local factors, independently of age. Ligature-induced periodontitis (LIPD) induced elevated gingival senescence biomarker expression, particularly in old mice, with a ~4-fold increase in SA-b gal (Fig. 1B and C), ~12-fold increase in p16 ^INK4A^ (Fig. 1D), ~4-fold increase in TNFa (Fig. 1E), and ~7-fold increase in IL-1β (Fig. 1F). Oral gavage with senescence inducer Pg [14] further augmented SA-b gal expression in the gingiva of the young, equivalent to that observed in old mice, suggesting “premature” senescence. Rapamycin ablated the gingival senescence response. Further revealed was consistent escalation (~47%) of bone loss at ligated site in the old mice (Fig. 2A-C), suggesting more rapid progression in response to ligature placement. Pg oral gavage increased bone loss by ~40% in young mice, equivalent to that in old mice. The senolytic agent rapamycin (Rap) ablated Pg-mediated bone loss in young and old mice. No significant differences were observed in bone loss between male and female mice (Supplementary Fig. 2). Collectively these data support the hypothesis that alveolar bone loss in this model involves senescence induction in response to local precipitating factors and is exacerbated by advanced age.

### Senescent DCs and T cells induced in gingiva and draining lymph nodes (LN) by LIPD and Pg

**Figure 3.**
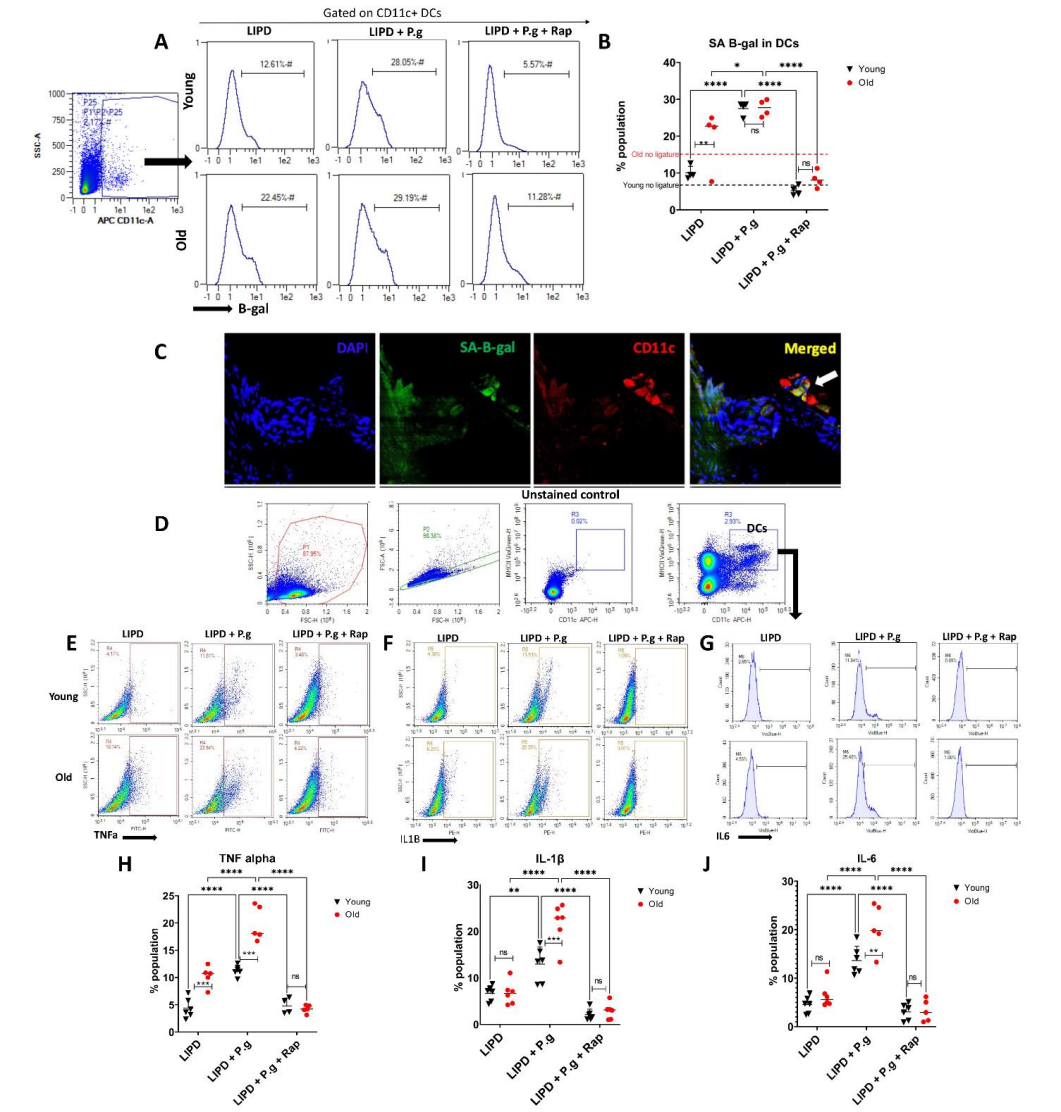
Senescent DCs in gingiva and LN. Gingiva and LN of young and old mice subjected to LIPD, with or without Pg gavage and senolytic agent rapamycin (Rap) were excised, cells isolated, labeled and analyzed by FACS for (A-B) fluorescent SA-B-gal^+^ CD11c^+^DCs (N=6 per group, gingival tissues pooled and n= 4 technical replicates.) (C) 3-color IF confocal microscopy of SA-B-gal+ CD11c+ DCs in gingiva from Pg-induced PD mice merged panel showing co-localization (yellow) of B-gal (green) with DCs(red) (D) Gating strategy for DCs in LN. (E,F,G) Representative FACS scattergrams showing intracellular staining for TNFα, IL-1β, and IL-6 in CD11c^+^ MHCII^+^DCs in LN. (H,I,J) Summary analysis of FACS scattergrams (N=6 per group). Two-way ANOVA and Tukey post-hoc multiple comparison test used (* p<0.05, **p<0.001, ***p<0.0001).

To further guide the immune senescence studies, we analyzed blood samples from a small cohort of older (n=3, mean age 68.3+2.8) and younger (n=3, mean age 24+5.5) healthy adult donors for age-vulnerable immune cell subtypes [12, 13]. Significant age-related changes in CD57+CD28- putative senescent T cells, (S3) non-classical monocytes (S4), late memory B cells (S5), classical myeloid DCs (S4) and CD19+ B cells (S5) were observed in the older cohort. These data, combined with evidence for a role of myeloid DCs and Th17 CD4+ T cells in advanced bone loss in humans [18] and mice [17], directed our focus on senescence biomarkers in DCs and T cells in gingiva and LN of young/old mice. Our data indicate that advanced age alone increased expression of SA-b-Gal in gingival CD11c+ DCs from 6.73% (young) to 16.7%) (old), represented as dotted lines in Fig. 3B. Implementation of LIPD further increased % SA-b-Gal^+^ DCs to 12.6% (young) and 22.45% (old) (Fig. 3 A and B). Senolytic/senomorphic agent rapamycin ablated influence of age and Pg on the SA-b-Gal response in DCs (Fig. 3A and B). Gingiva of mice subjected to LIPD were stained for SA-b-Gal (green) and CD11c (red) and analyzed by confocal microscopy (Fig. 3C). Merged channels demonstrate colocalization (yellow) of SA-b-Gal with CD11c+ DCs in gingiva *in situ* (white arrows) corroborating ex vivo DC isolation and FACS analyses (Fig. 3A). LN DCs of old and young mice subjected to LIPD were analyzed by FACS for known biomarkers of the SASP, intracellular TNFa, IL-6 and IL1b (Fig. 3D-J). A significant elevation in TNFa in LN DCs of old mice was observed (Fig. 3 E and H). Pg oral gavage increased TNFa, IL1b and IL6 response in LN DCs, from young or old mice, which was ablated with Rap (Fig. 3E-J).

### Induction of IL-17+, CD28^-^CD57^+^ senescent T cells in draining lymph nodes (LN) of old mice by LIPD and Pg

**Figure 4.**
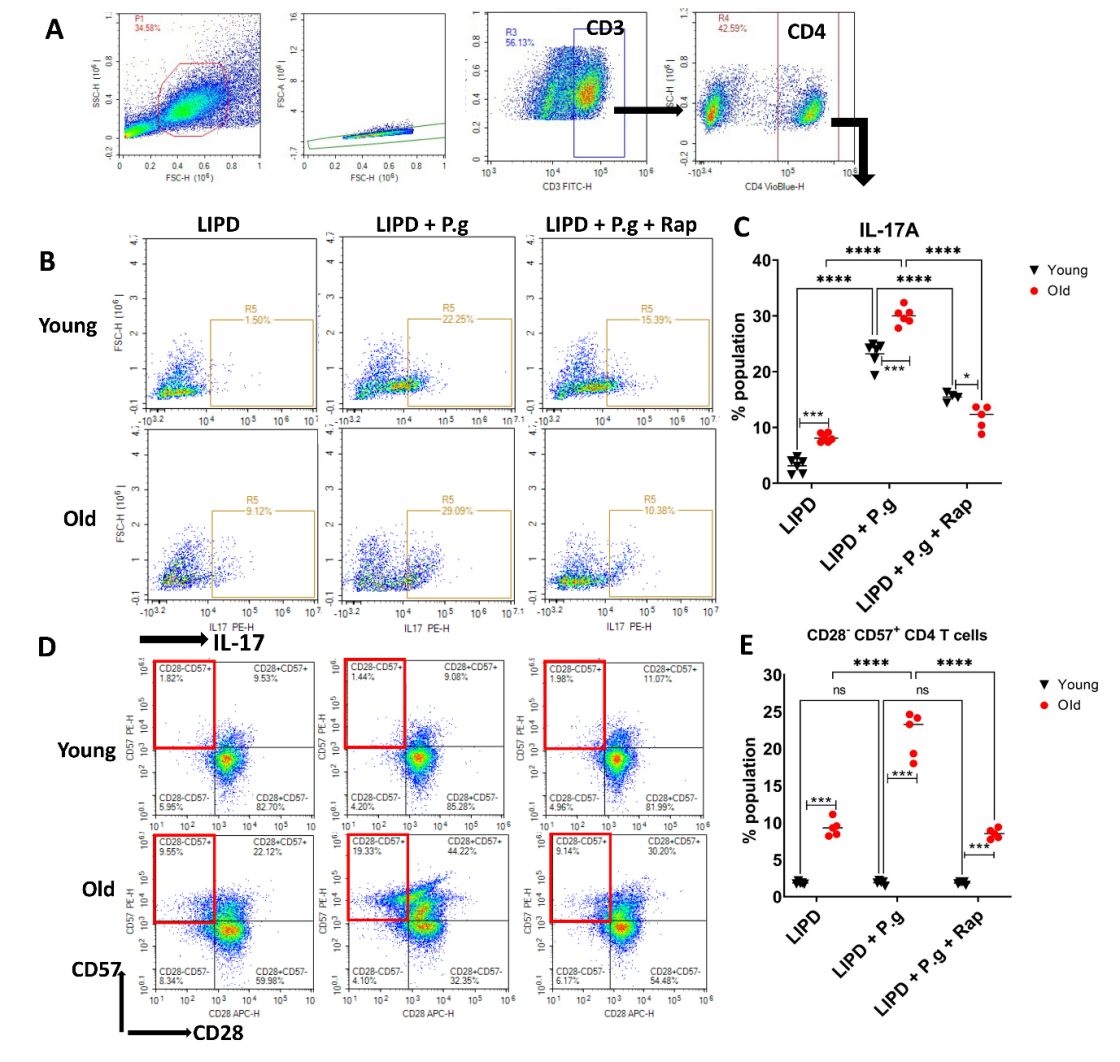
Th17 and senescent T cells in LN induced by PD. (A) FACs scattergrams showing gating strategy for CD4+ T cells in LN. (B and C) IL-17^+^ CD4T cells and (D+E) CD28^-^CD57^+^expression in CD4T cells (N=6 per group, Two-way ANOVA and Tukey post-hoc multiple comparison test used (* p<0.05, **p<0.001, ***p<0.0001).

Unregulated Th17 cells promote inflammatory bone loss in mice through RANK-L-mediated osteoclastogenesis [17]. Recent evidence suggests a potential role of senescent T cells in age-related inflammatory diseases [28]. Here we analyzed the percentage of IL17^+^ and %CD28^-^CD57^+^ T cells [29] in draining LN of mice subjected to LIPD, in the presence or absence of Pg or Pg + rapamycin (Fig. 4). Observed was an elevation of IL-17^+^ (Fig. 4B, 4C) and CD28^-^CD57^+^ senescent CD4^+^ T cells (Fig. 4D, E) in LN of old mice vs. young mice, as induced by LIPD. Moreover, Pg gavage provoked a strong IL-17+T cell response in young mice and further exacerbated the senescent CD28^-^CD57^+^ CD4^+^T response in LN of old mice.

### Gingival injection of Pg-induced DC exosomes promotes senescence and inflammatory bone loss

**Figure 5.**
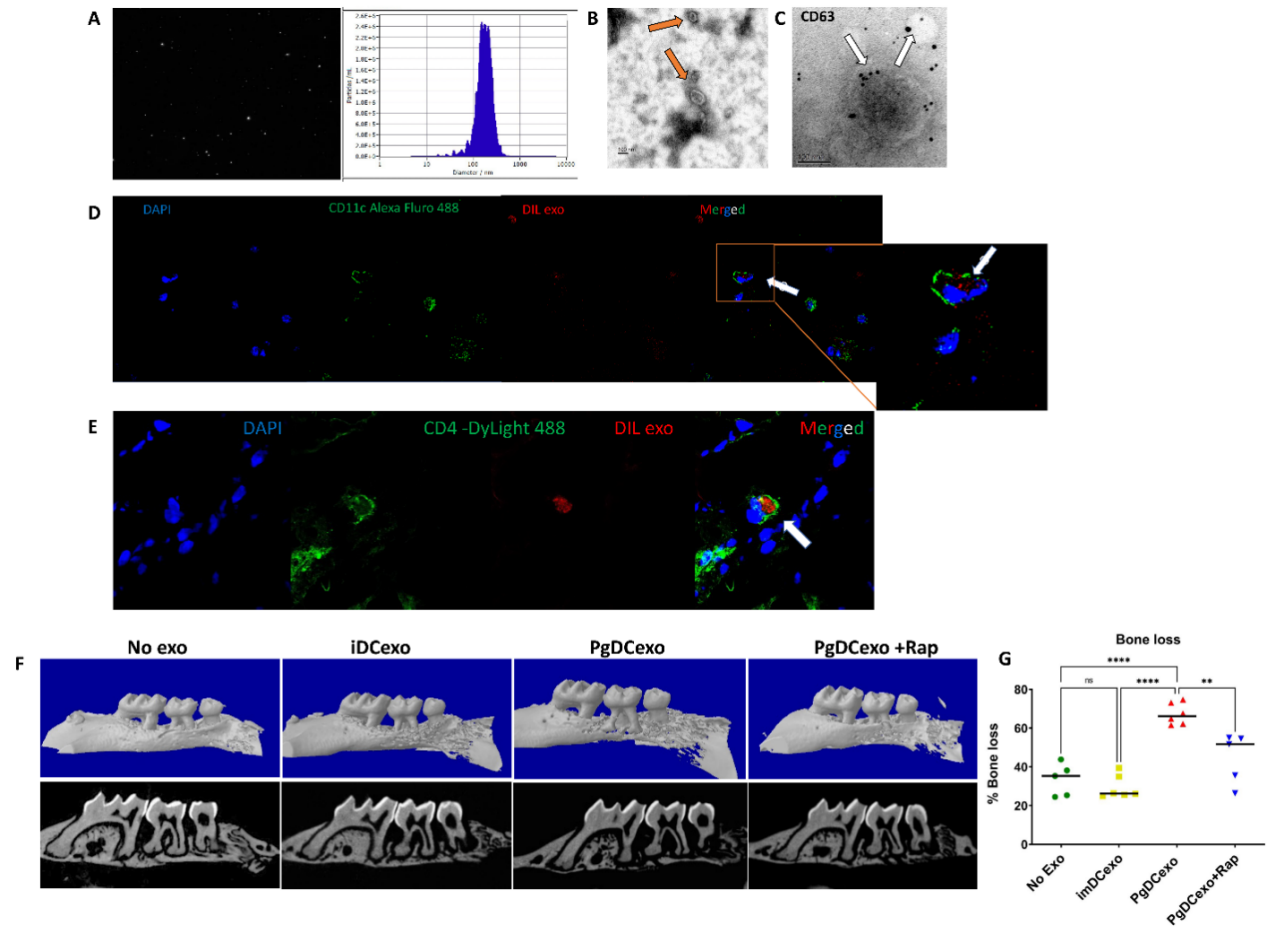
Exosomes derived from DC infected with Pg are internalized into DCs and CD4 T cells in situ and promote alveolar bone loss. (A) Nano tracking analysis (NTA) to determine Exo number and size distribution in nanometer (nm). (B) SEM showing characteristic Exo morphology (orange arrows) (C) Immuno-gold TEM showing EXO marker, tetraspanin CD63 (D-E) Confocal microscopy images showing the uptake of Dil-labeled PgDCexo (red) in (D) CD11c^+^ DCs (green) and in (E) CD4^+^ T cells (green) in gingival tissues of mice. Nuclei were counterstained with DAPI (blue). (F) MicroCT analysis showing bone loss in young mice induced by gingival injection of PgDCexo but not iDCexo (N=6 per group).

**Figure 6.**
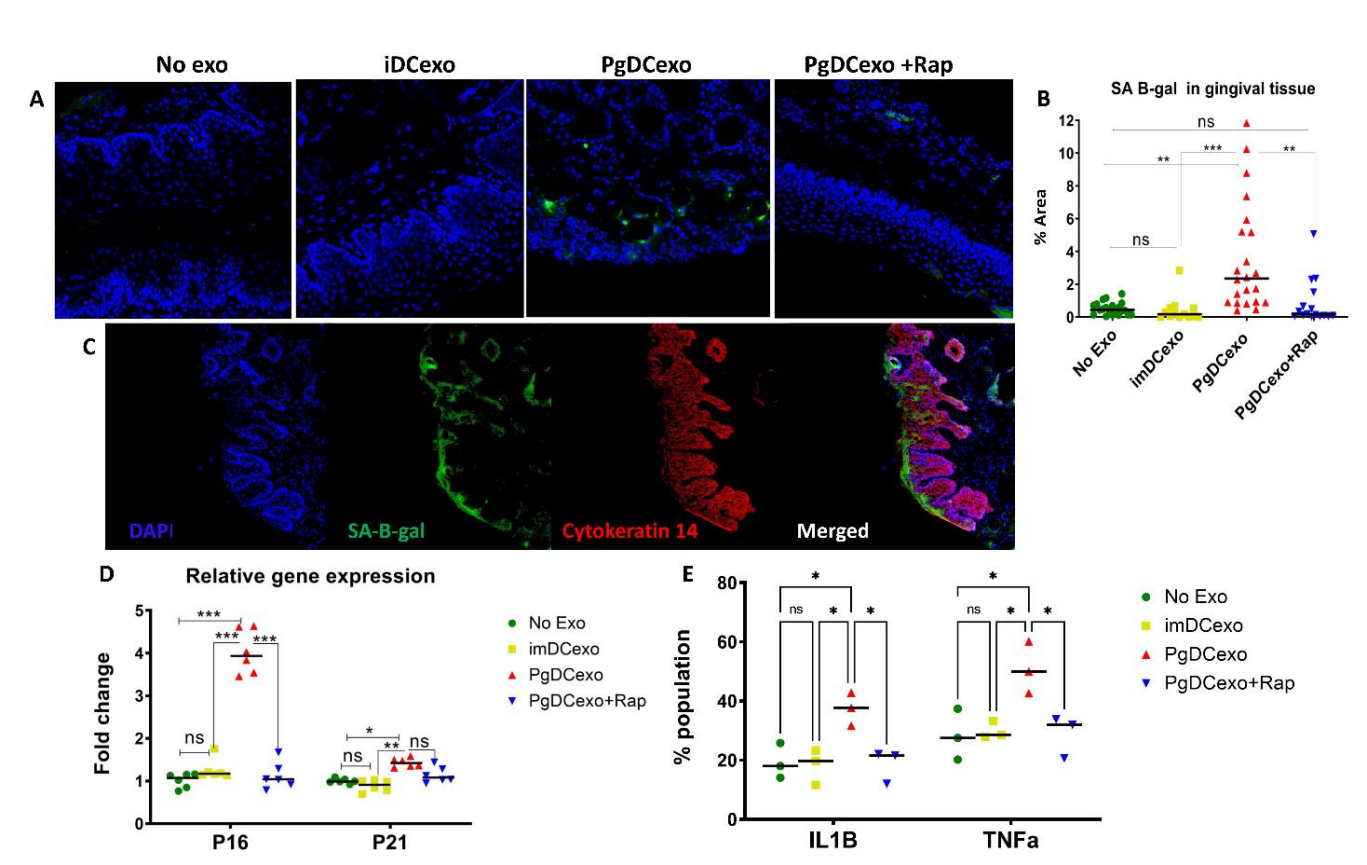
Intragingival injection of exosomes promotes paracrine senescence in gingival tissues (A) Representative confocal microscopy images showing SA-B-gal expression in the gingiva (green) and counterstained with DAPI (blue) for nuclei. (B) Quantification of SA-B-gal expression plotted as % area of SA-B-gal^+^cells using image J software (N=4 per group, multiple random images were taken per slide). (C) Immune fluorescence confocal microscopy of SA-B-gal (green) expression in keratinocytes labeled with Cytokeratin 14 (red) in the gingiva of mice injected with PgDCexo. In gingiva, PgDCexo induce increase in gingiva of: (D) mRNA relative gene expression (relative to no exo control group) of p16 ^INK4A^ and p21Waf1/Clip1 by qPCR analysis (N=6 per group, gingival tissues pooled and n=6 technical replicates) (E) protein expression of IL-1b and TNFa by FACS analysis (Scattergrams shown in S2) (N=6 per group, gingival tissues pooled and n=3 technical replicates). One way ANOVA and Tukey post hoc multiple comparison test used (*p<0.05, **p<0.001, ***p<0.0001) ImDCexo = exo from un-infected immature DCs (control), PgDCexo= exo from DCs infected with Pg.

Pg invasion of bone marrow derived DCs in vitro has been shown to induce senescence of host DCs, coupled with increased DC secretion of exosomes enriched in SASP-related inflammatory cytokines [14]. Here exosomes secreted by DCs upon Pg invasion (=PgDCexo) were purified, enumerated (Fig. 5A and B) and examined for correct phenotype by SEM (Fig. 5B) and immunogold TEM (Fig. 5C). Purity of PgDCexo (~99%) and lack of significant Pg outer membrane vesicles (OMV) was shown previously by our group [14]. PgDC exo and control iDCexo were injected intragingivally (S6) at the site of ligature placement, as we described [17]. The uptake of PgDCexo by DCs was monitored as previously documented by our group *in-vitro* [14] and is confirmed in the current study in-vivo in the gingival tissues of mice (Fig. 5D and E). Dil-labeled exo was observed internalized by CD11c^+^ DCs and CD4^+^ T cells in situ in the lamina propria of gingival tissues in mice. Moreover, PgDCexo induced a 2-fold increase in bone loss, relative to iDCexo (Fig. 5F, 5G); furthermore, this increased SA-b-Gal expression in gingival tissues (Fig. 6A and B) which was ablated by rapamycin. Histological sections of gingiva (Fig. 6C) revealed that PgDCexo also induced SA-b-Gal staining of epithelium. Moreover, p16 ^INK4A^ and p21 (Fig. 6D) mRNA were increased in whole gingival cells from mice injected with PgDCexo compared to control animals treated with iDCexo. Examination of IL1b and TNFa proteins by FACS analysis revealed a significant increase induced by PgDCexo. (Fig. 6E) Histograms are shown in supplemental data (S6). Collectively, these in vivo data strongly support our hypothesis that Pg-induced DC exosomes promote potent immune senescence.

### PgDCexo surmount senescence resistance of LN T cells from young mice

**Figure 7.**
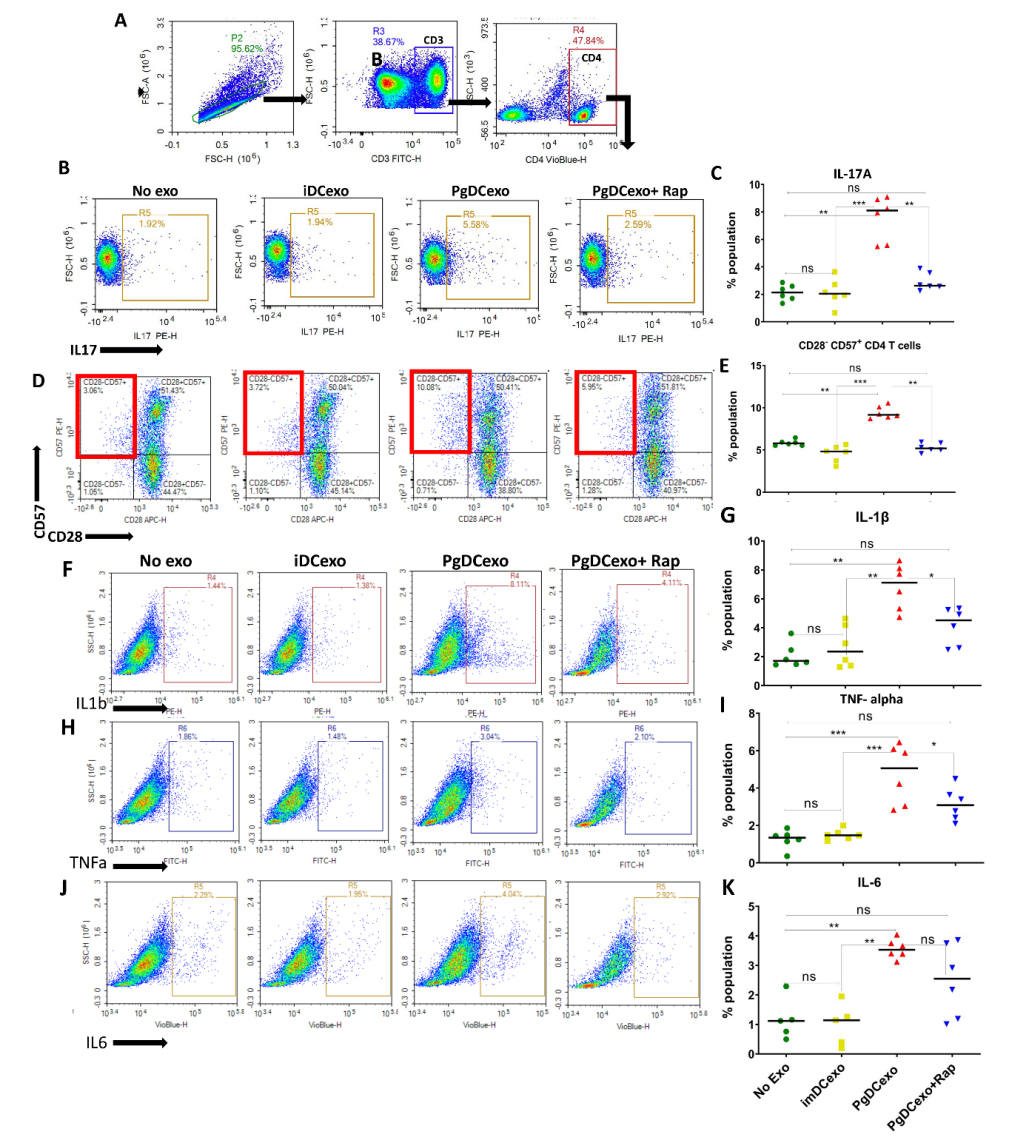
Intragingival injection of exosomes promotes Th-17^+^, senescent CD4^+^CD28^-^CD57^+^ T cells and inflammatory profile in DCs in LN. Cervical lymph nodes of young mice subjected to LIPD and injected with PgDCexo +/_ Rap or ImDCexo (control group) were harvested, cells isolated, labeled and analyzed by FACS (A) Gating strategy for T cells in LN (B, C) %IL17a^+^ CD4 T cells (N=6 per group) (D, E) % CD28-CD57^+^ senescent CD4 T cells (N=6 per group). (F-K) FACS Intracellular staining showing inflammatory cytokine profile of DCs in LN; (F, G) IL-1β, (H, I) TNFa, and (J, K) IL-6, cells gated on CD11c^+^ MHCII^+^ (gate not shown) (N=6 per group). One way ANOVA and Tukey post hoc multiple comparison test used (*p<0.05, **p<0.001, ***p<0.0001) ImDCexo = exo from un-infected immature DCs (control), PgDCexo= exo from DCs infected with Pg.

CD28^-^CD57^+^ T cells play a pathogenic role in age-related and osteolytic diseases [30]. Both CD28^-^CD57^+^ CD4 and CD8 T cells have been associated with aging and recognized as markers for T cell senescence in humans [29] (Supplementary Fig. 1) and in mice [31, 32]. Our data suggested that CD4 T cells from LN of young mice were more resistant to senescence induction by LIPD and Pg (Fig. 4D and E). PgDCexo surmounted the senescence resistance of T cells in young mice, inducing ~3-fold increase in senescent %CD28^-^CD57^+^ CD4^+^ T cells (Fig. 7D, 7E) and %Th17 effectors (Fig. 7B and C) compared to iDCexo. SASP-related inflammatory cytokines were also potentiated in DCs by gingival PgDCexo injection (Fig. 7F-K). These results suggest that Pg-induced DC exosomes are a potent mechanism for transmitting and amplifying paracrine senescence in bystander normal cells.

## DISCUSSION

The aging adult population is growing rapidly in the USA and it is expected that by 2040 the number of adults ≥ 65 years of age will have increased by about 50% [33]. Inflammaging, or elevation in baseline inflammation with advanced age, is a major contributor to the pathogenesis of many age-related diseases, such as type 2 diabetes, heart disease, cancer, Alzheimer’s disease, and PD [6, 34]; however, the underlying mechanisms are poorly understood. In the current study we show that immune senescence plays an influential role in alveolar bone loss in the experimental PD model. Local factors, including ligature placement and Pg oral gavage, compounded by advanced age, were shown to induce immune senescence and alveolar bone loss in this model, which were ablated by senolytic agent rapamycin (Fig. 1-2).

In the current study we show that the LIPD and microbial insult with Pg exacerbate physiologic immune senescence, contributing to inflammatory bone loss. These data strongly suggest that the presence of senescent immune cells and constitutive inflammaging associated with advanced age render the host more vulnerable to the influence of local etiologic factors, in this case the presence of ligatures and the dysbiotic pathogen Pg, thereby favoring progression of the disease. It is also plausible that senescent cells become more permissive to bacterial invasion, enhancing Pg uptake and increasing bacterial load. Salmonella uptake by senescent fibroblast was enhanced due to senescence-induced increase in caveolin-1 expression [35]. Bleomycin- or H2O2- induced senescence in mice enhanced the levels of p16, and pro-inflammatory cytokines in the lungs, as well as the susceptibility to pneumococcal infection where senescent cells were more permissive to bacterial infection in vitro [36]. In line with these studies, there was an increase in susceptibility to Pg infection in old mice as evidenced by enhanced bacterial levels detected on the teeth surfaces [37] from aged mice. It can be surmised that age-related immune senescence and its associated SASP in mice produce an environment conducive to inflammaging, enhanced bacterial invasion and contribute to alveolar bone degeneration accelerated by oral dysbiosis. The significance of these findings, i.e. for human disease, are evident in the NHANES database, showing that older adults have a higher prevalence of PD [1], and that the disease progresses with aging. Taken together, immune senescence and its associated SASP can be considered as potential therapeutic targets for mitigation or even reversal of the PD.

Rapamycin is an FDA approved immune suppressant drug [38] with antiaging properties [39, 40]. Rapamycin hinders senescence in human gingival fibroblasts [40] and mouse fibroblasts [39] reportedly by inhibition of oxidative stress [40] and Nrf2 activation [39] respectively. Systemic administration of rapamycin to old mice to modulate alveolar bone levels have previously been reported [41], though microbial and other local factors were not considered. Microbial dysbiosis elicits a bone destructive inflammatory response in the murine PD model [37]. We therefore implemented ligatures (i.e., LIPD) to augment endogenous dental biofilm accumulation, with addition of human dysbiotic pathogen Pg to better emulate plaque-induced PD in humans [42]. Moreover, we mitigated off-target effects of systemic rapamycin by intra-gingival injection of 10 µM, [25] three times weekly around ligated teeth. The ability of rapamycin to enhance bone formation [43] through inhibition of osteoclastogenesis [41] has prompted its applications for senile osteoporosis [44] and arthritis in mice [25]. Commensurate with inhibition of LIPD-induced bone resorption by rapamycin, there was decline in immune senescence and SASP production in both young and old mice, supporting our hypothesis, with caveats. Rapamycin inhibits mTOR, thereby promoting autophagy, pathways also implicated in senescence and age-related disease [45]. However, Pg inhibits autophagy in DCs promoting its survival [26] and its dissemination to distant sites [46]. Autophagy declines with aging [47] with an inverse relationship between autophagy and senescence documented [48]. Thus, a gene targeted approach is warranted to more definitively identify the causal role of immune senescence in PD.

The SASP, a characteristic feature of senescence, consists of secreted exosomes and inflammatory cytokines [4, 49]. Growing evidence supports a pathologic role of exosomes in immune senescence, inflammaging and age-related diseases [24], with secretion coupled to senescence induction [50]. Exosomes derived from infected cells have previously shown to carry bacterial RNA and protein, and are purported to spread infection and disease [51]. *Pg*-infected DCs secrete more exosomes, which transmit immune senescence to bystander immune cells in vitro, thus amplifying senescence [14]. Here, Pg-induced DC exosomes (PgDCexo) were injected intra-gingivally to assess influence on alveolar bone loss (Fig. 5) and immune senescence (Fig. 5-7). PgDCexo promoted an increase in gingival SA-b-Gal, p16, p21 expression and increase in IL-17A and CD28^-^ CD57^+^senescent CD4 T cells and SASP-related cytokines in gingival tissues and draining lymph nodes of young mice, with commensurate increase in bone loss. Worthy of note is the ability of PgDCexo to surmount the senescence resistance of T cells in young mice suggesting that Pg-induced DC exosomes are a potent mechanism for transmitting and amplifying paracrine senescence in bystander normal cells. PgDCexo contain TNFα, IL1β and IL6 [14] and express the adhesive Pg Mfa1 fimbrial protein [14], through whether or not the anti-autophagy/apoptosis functions of *Pg* Mfa1 fimbriae [26] are retained in exo is unclear. Additionally, these exosomes are enriched in age-related, anti-apoptosis/anti-autophagy miRNAs as shown previously by our group [14]. Exosomes protect their protein cargo against proteolytic degradation [17]. Presence of gingipains or other forms of bacterial antigens in PgDCexo is yet to be elucidated. Moreover, the fate of PgDCexo after injection is not yet known, though we have shown that intra-gingivally injected therapeutic exosomes from DC had high affinity for inflamed gingival sites and were taken up by both DCs and T cells in situ [17]. This is particularly important due to ability of exosomes to travel to distant sites [52] and cross blood brain barrier [53]. This information can explain how oral pathogens such as Pg may have a systemic impact in other age-related chronic inflammatory diseases such as Alzheimer’s disease and cardiovascular diseases.

We conclude that local factors that exacerbate oral biofilm formation or that cause a dysbiosis promote premature immune senescence in gingival tissues and draining lymph nodes of young mice and exacerbate physiologic senescence and inflammatory response in old mice. These activities are commensurate with progression of degenerative alveolar bone loss. The ability of Pg to activate the DC SASP, releasing pathologic exosomes capable of promoting and amplifying senescence in normal bystander cells *in paracrine*, appear to be particularly influential in induction of alveolar bone loss.

## Supplementary Materials

The Supplementary data can be found online at: www.aginganddisease.org/EN/10.14336/AD.2022.0623.

## Data Availability

The raw data supporting the findings of this study will be made available by the authors, upon reasonable request.
