# Supplementary material for: Microbially-Induced Exosomes from Dendritic Cells Promote Paracrine Immune Senescence: Novel Mechanism of Bone Degenerative Disease in Mice
Source: Aging Dis. 2023 Feb 1;14(1):136–51. doi: 10.14336/AD.2022.0623 (PMC9937696; doi:10.14336/AD.2022.0623)
Supplement: Supplementary file 1 — The Supplementary data can be found online at: www.aginganddisease.org/EN/10.14336/AD.2022.0623. [file AD-14-1-136-s.pdf]

SUPPLEMENTARY DATA

Microbially-Induced Exosomes from Dendritic Cells Promote Paracrine Immune Senescence: Novel Mechanism of Bone Degenerative Disease in Mice

Ranya Elsayed[1], Mahmoud Elashiry[1], Yutao Liu[2], Ana C. Morandini[1,3], Ahmed El-Awady[1], Mohamed M. Elashiry[4], Mark Hamrick[2], Christopher W. Cutler[1*]

© 2022. Elsayed R et al. Published online at http://www.aginganddisease.org/EN/10.14336/AD.2022.0623

SUPPLEMENTARY DATA

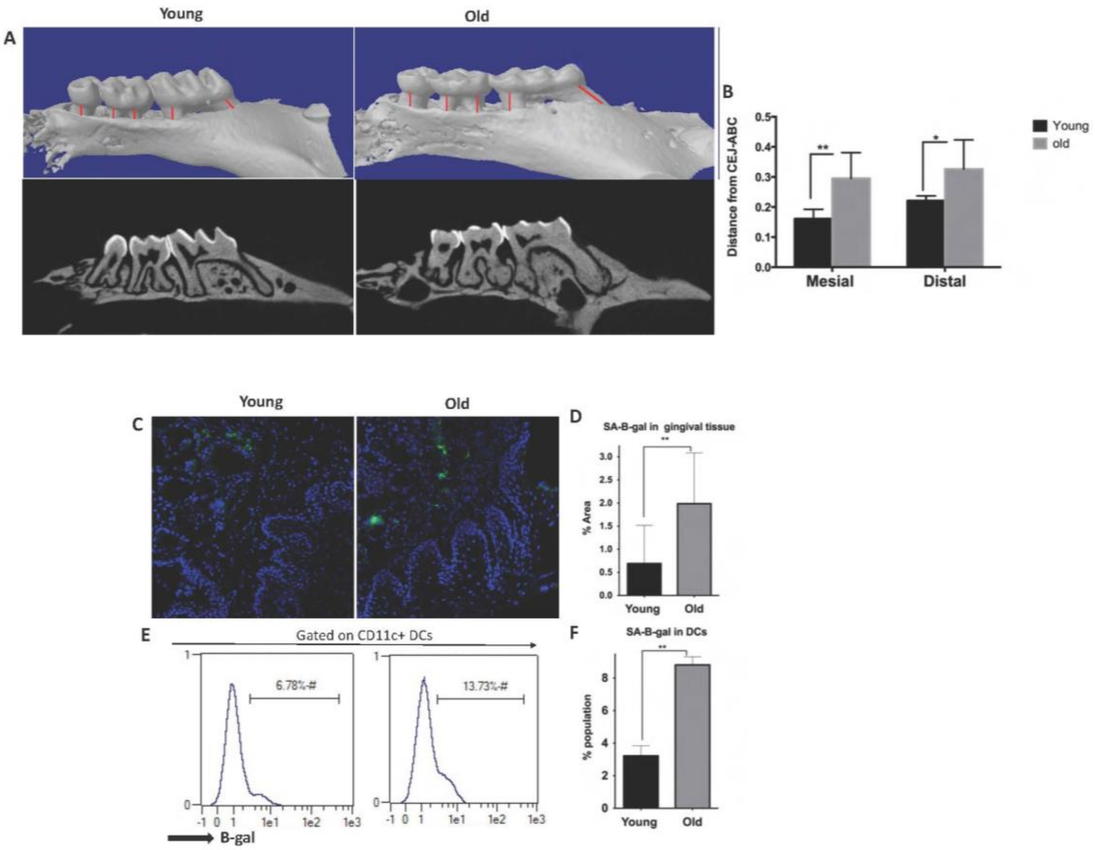

Supplementary Figure 1. Old mice show physiological baseline senescence in gingival tissues and alveolar bone resorption compared to young mice. Gingiva of control young and old mice which received no treatment was stripped, and frozen sections were used for detection of SA-B-gal using fluorescent senescent B-gal probe. Gingival tissues were pooled, cells isolated, labelled, and analyzed by FACS for fluorescent SA-B-gal expression in DCs (A) Representative micro-CT generated 3-D images of maxilla with teeth; red lines showing distance from CEJ to ABC. (B) Bar graphs of linear measurements of the distance between CEJ-ABC of the mesial and distal roots of upper second molar of young and old mice (n=6). (c) Representative confocal microscopy images showing SA-B-gal expression in the gingiva (green) and counterstained with DAPI for nuclei. (D) Quantification of SA-B-gal expression plotted as % area of SA-B-gal $^{+}$cells using image J software. (E, F) FACS analysis showing SA-B-gal expression in CD11c^{+} DCs in gingival tissues of young and old mice.

© 2022. Elsayed R et al. Published online at http://www.aginganddisease.org/EN/10.14336/AD.2022.0623

SUPPLEMENTARY DATA

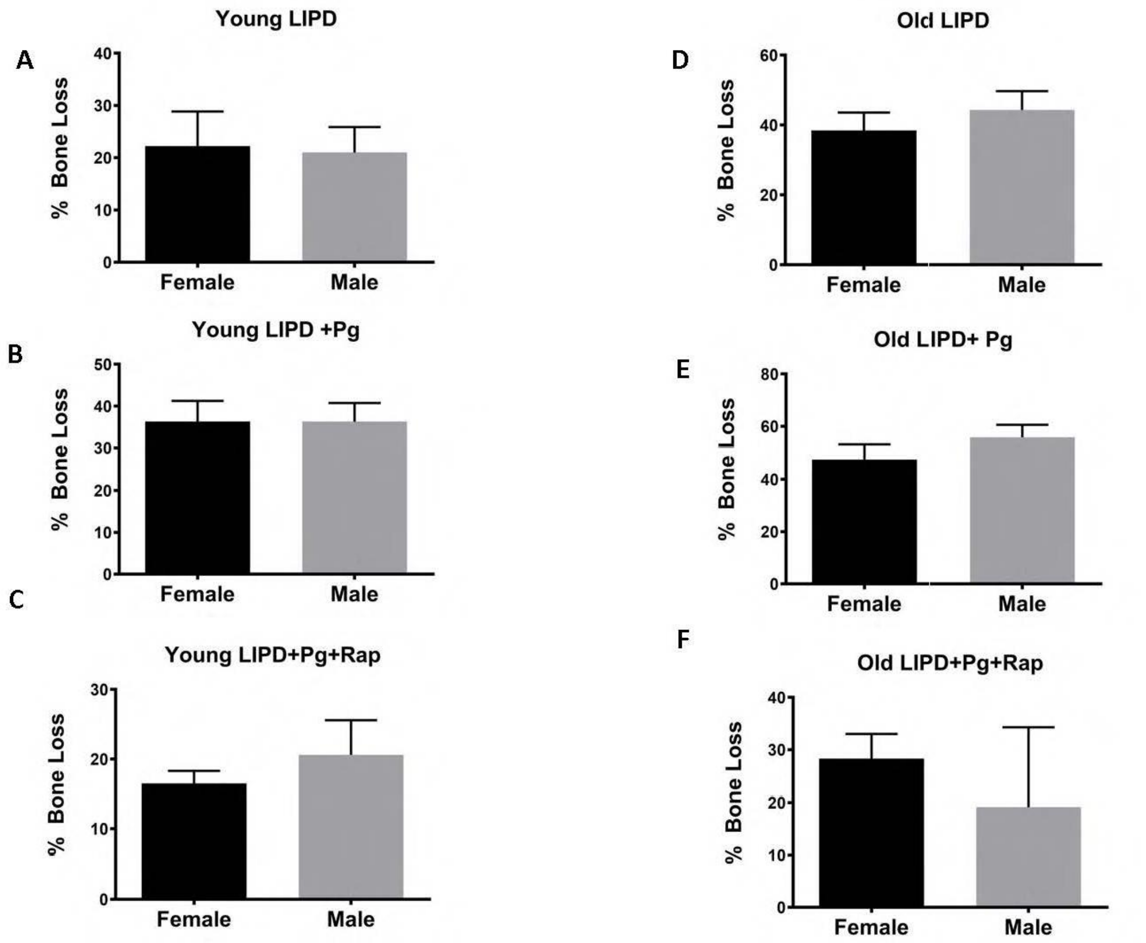

Supplementary Figure 2. Gender differences in% bone loss with ligature induced periodontitis (LIPD) and *P.gingivalis* oral gavage (Pg)+/rapamycin (Rap) in young and old mice.

© 2022. Elsayed R et al. Published online at http://www.aginganddisease.org/EN/10.14336/AD.2022.0623

SUPPLEMENTARY DATA

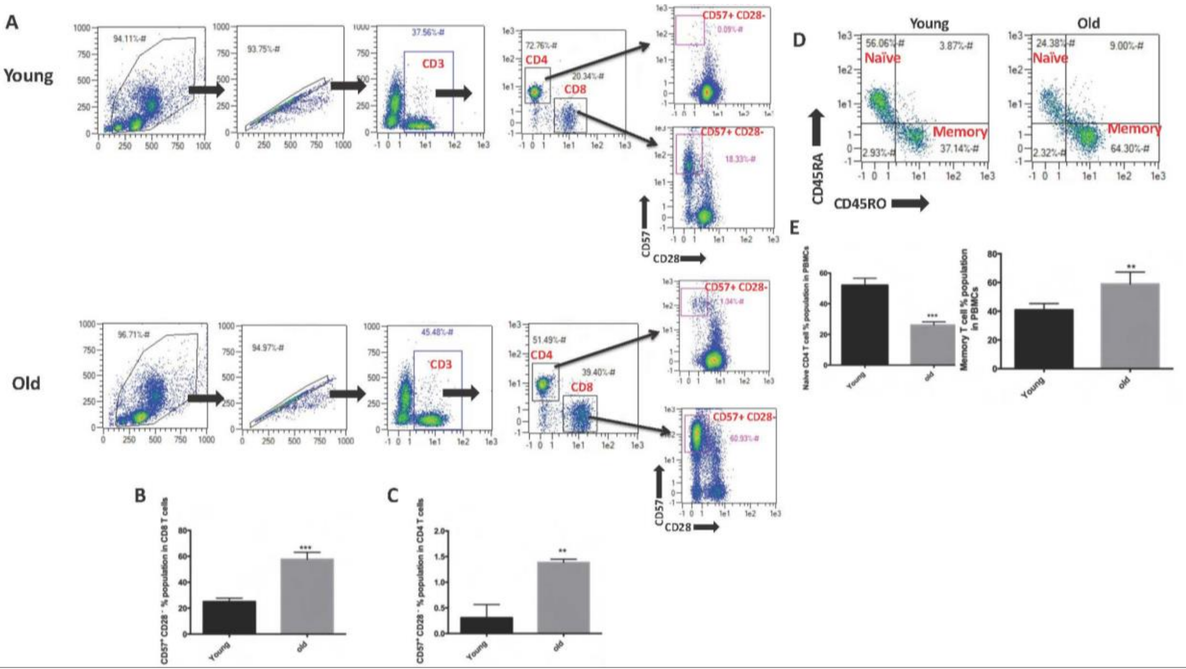

Supplementary Figure 3. Increase in senescent CD8 and CD4 T cells in older subjects. Buffy coats from young (n=5, mean age 24±5.5) and old (n=5, mean age 68.3±2.8) donors were obtained from Community Blood Center (periodontal status unknown). (A) Scattergrams and gating strategy for %CD3, CD4, CD8 and CD57+CD28- subsets are shown. (B) Means of %CD57+CD28- CD8+ (C) and CD4+ T cells are shown. *P<0.05, Students T test.

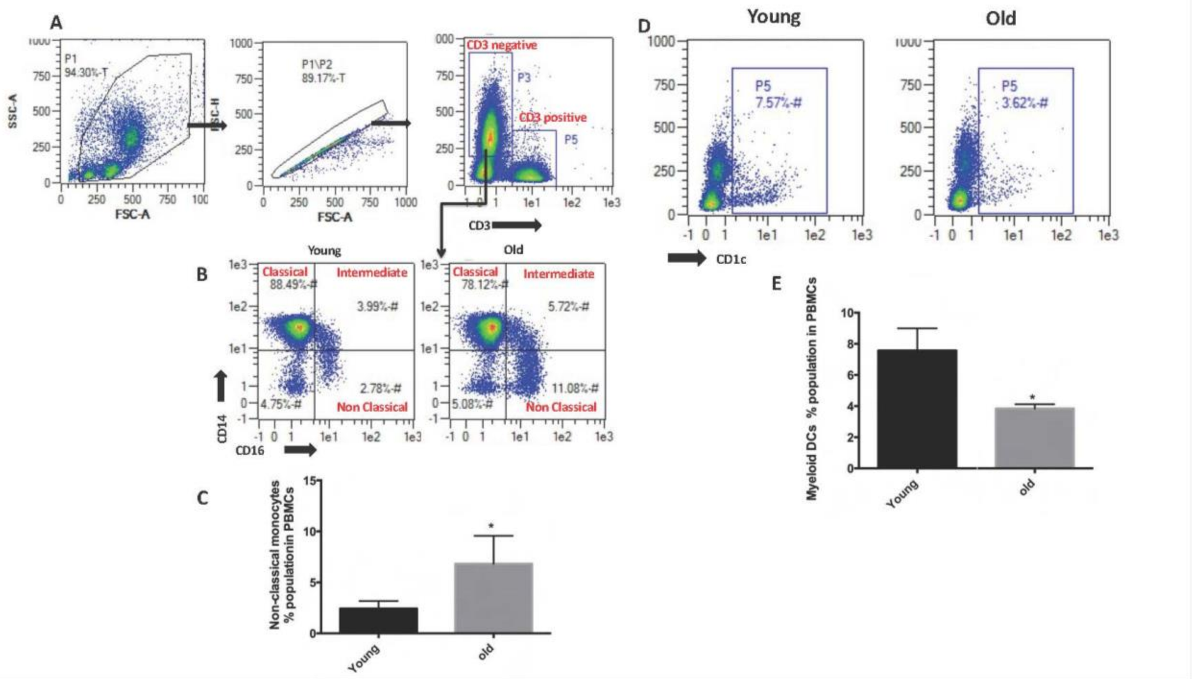

Supplementary Figure 4. Increase in non-classical monocytes and decrease in myeloid DCs with advanced age. (A) Scattergrams of CD3- monocyte subsets, showing (B&C) increase in non-classical monocytes and (D&E) decrease in CD1c+ DCs in old cohort.

© 2022. Elsayed R et al. Published online at http://www.aginganddisease.org/EN/10.14336/AD.2022.0623

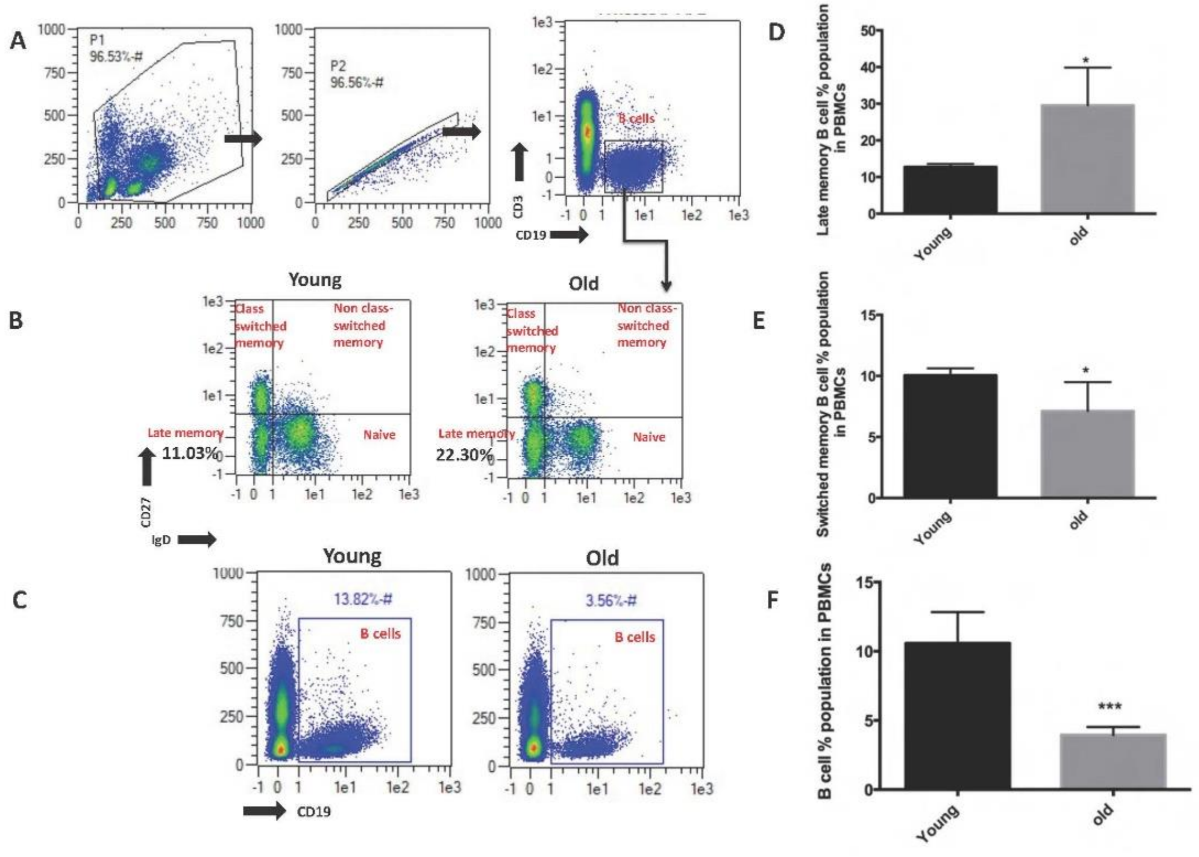

Supplementary Figure 5. **Figure 16. Senescent B cell subsets in older subjects.** (A) Representative scattergram of CD19+CD3- B cells and (B) Naïve (IgD+CD27-), unswitched memory (IgD+CD27+), switched memory (IgD-CD27+), and late memory (IgD-CD27-) in old and young cohort. (C) CD19+CD3- B cells (D-F) Summary bar graphs showing (D) mean % late memory and (E) % switched memory B cells and (F) %CD19+CD3- B cells.

© 2022. Elsayed R et al. Published online at http://www.aginganddisease.org/EN/10.14336/AD.2022.0623

SUPPLEMENTARY DATA

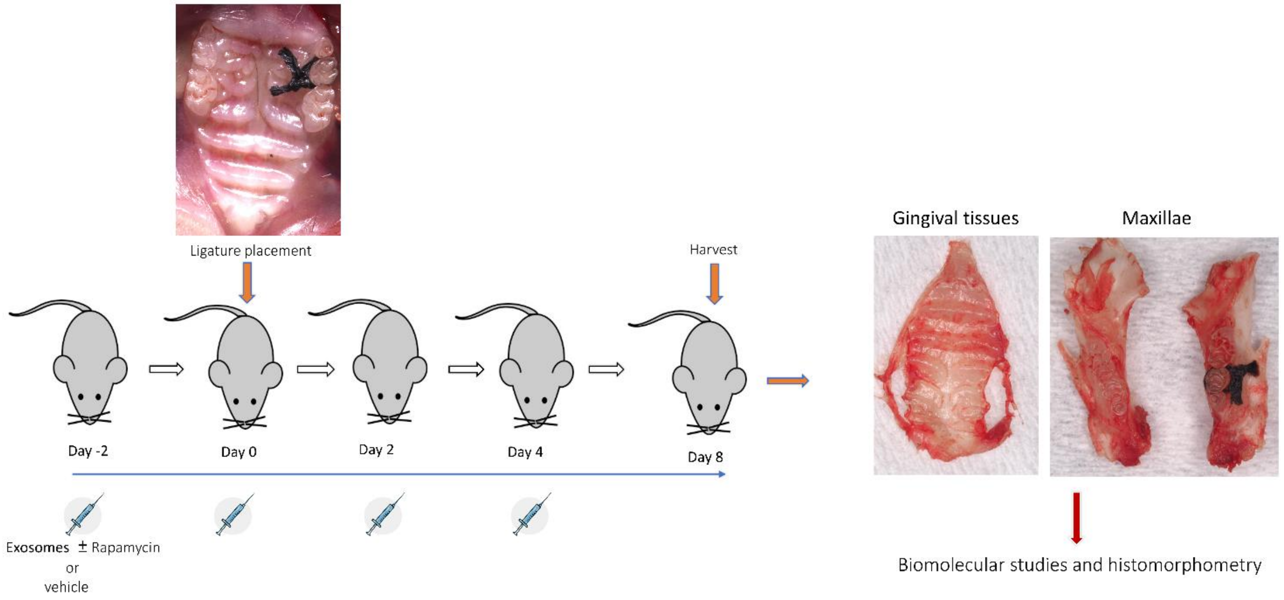

Supplementary Figure 6. Study design for intragingival injection of exosomes in young mice. A schematic diagram showing the animal study design.

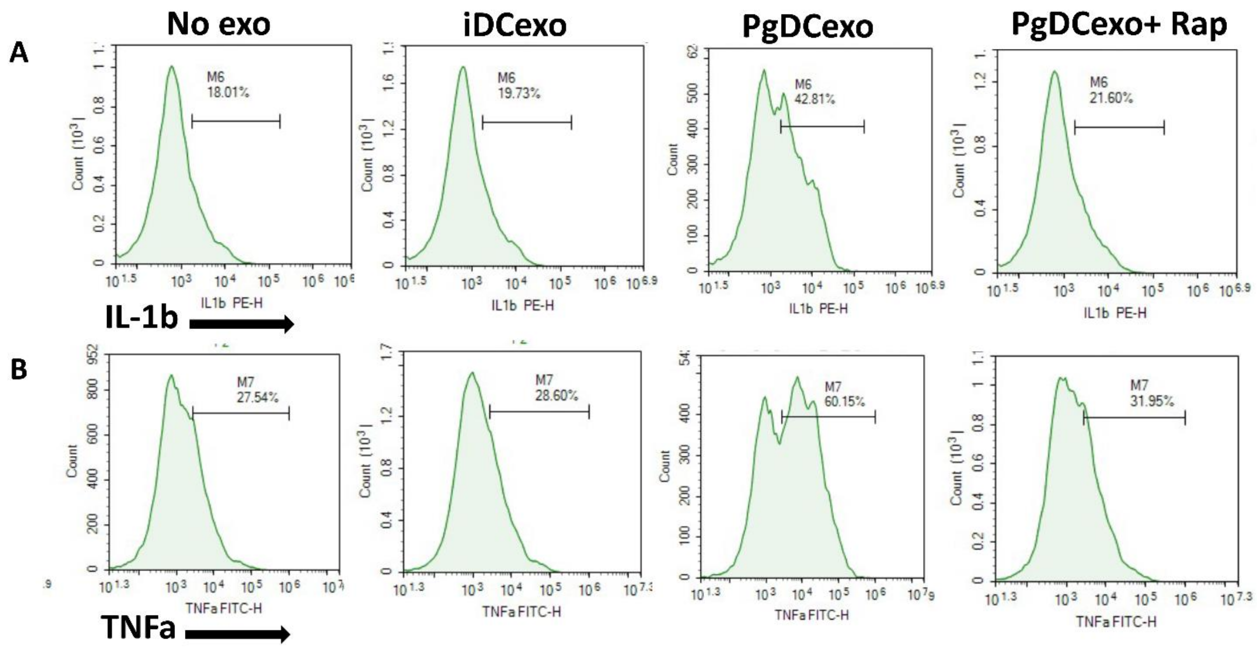

Supplementary Figure 7. Histograms showing protein expression of IL-1β and TNFα by FACS analysis from the gingiva of mice injected with PgDCexo or imDCexo +/- Rap.

© 2022. Elsayed R et al. Published online at http://www.aginganddisease.org/EN/10.14336/AD.2022.0623

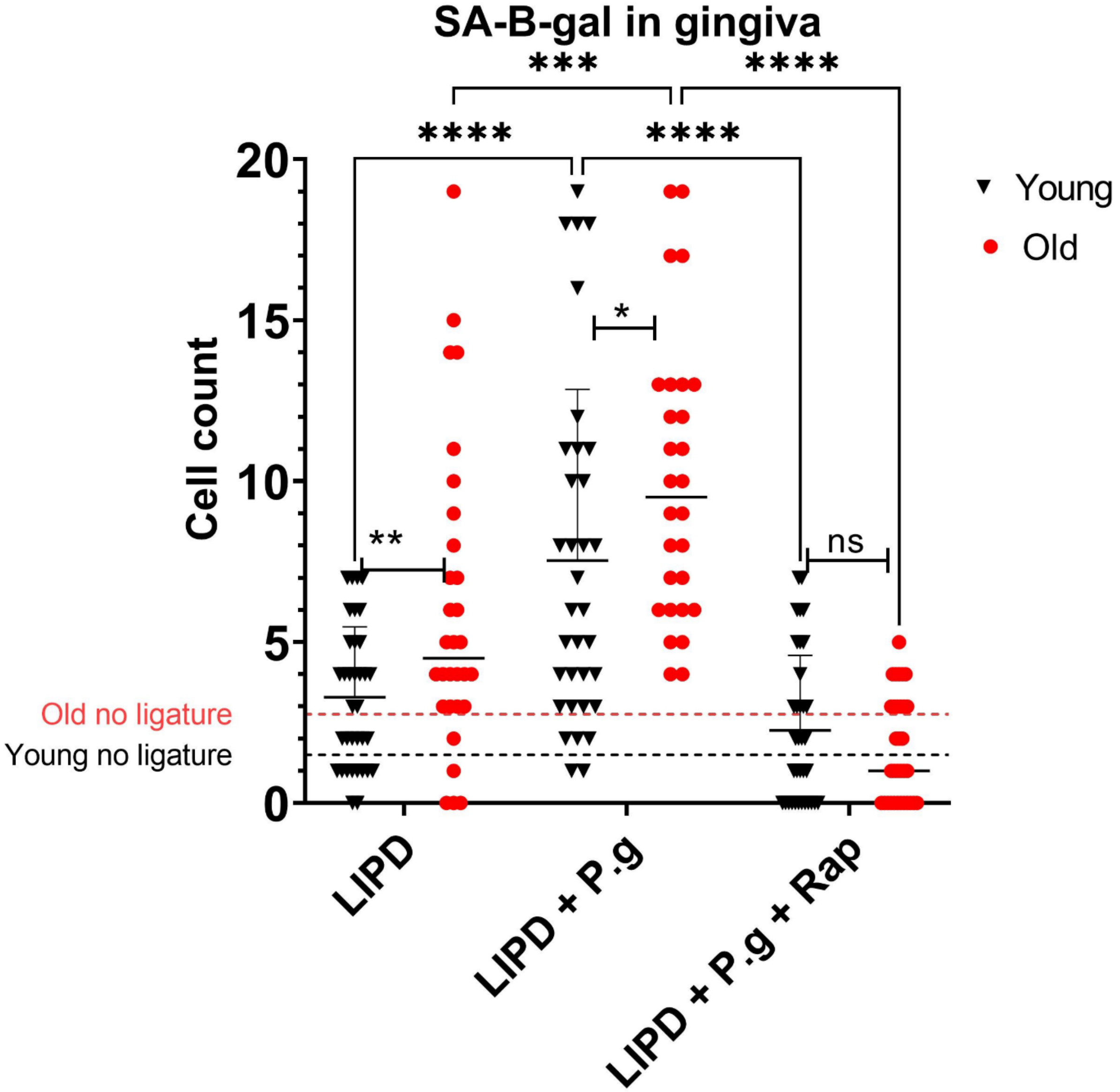

Supplementary Figure 8. **Quantification of SA-B-gal expression in gingival tissues.** Counting of SA-B-gal $^{+}$cells was done by a blind observer (N=4 per group with multiple random images taken per animal).

© 2022. Elsayed R et al. Published online at http://www.aginganddisease.org/EN/10.14336/AD.2022.0623
